# Supplementary material for: Geographical variation and associated factors of vitamin A supplementation among 6–59-month children in Ethiopia
Source: PLoS One. 2021 Dec 31;16(12):e0261959. doi: 10.1371/journal.pone.0261959 (PMC8719719; doi:10.1371/journal.pone.0261959)
Supplement: Attachment — Submitted filename: Response to Reviewers.docx [file pone.0261959.s001.docx]

We are very thankful for the journal, editorial office and reviewers who gave their time to improve this work. We tried to admit every point rose by the editorial office and reviewers unless there might be a misunderstanding of the points. The detailed responses are as follow.

**Reviewer** #1

**Comment**: L. 21: Capitalize “Vitamin” and use “that is” instead of “which are

**Response**: done…. Vitamin A has been one of the most important micronutrients that is necessary for the health of children

**Reviewer** #1

**Comment:** L. 36: delete “was”

**Response**: The distribution followed some spatial geo-locations **Reviewer** #1

**Comment:** L. 39: delete “have”

**Response**: Place of delivery 1.2(1-1.34), secondary and education 1.3 (1-1.6), media exposure 1.2(1.1-1.4), having work 1.4(1.2-1.5), and all visits of ANC positively influenced the distribution.

**Reviewer** #1

**Comment:** 41-42: The sentence is poorly constructed. Perhaps delete “was poor”.

**Response**: The distribution of vitamin A supplementation coverage was not random according to the data from the Ethiopian demographic health survey

**Reviewer** #1

**Comment:** L. 51: World Health Organization (WHO), vitamin A …

**Response**: World Health Organization (WHO)

**Reviewer** #1

**Comment:** L. 54: Delete world health organization

**Response**: In areas where vitamin A is a community problem, WHO recommends mass supplementation

**Reviewer** #1

**Comment:** Throughout: Do not capitalize vitamin A unless it is starting the sentence, i.e., L. 60, 154, 283

**Response**: unnecessarily capitalized vitamin A corrected

**Reviewer** #1

**Comment:** L. 68: Not sure what this phrase means.

**Response**:

**Reviewer** #1

**Comment:** L. 74: United Nations Children Fund

**Response**: Vitamin A is the known public health problem in the countries where preschool children were not targeted for the supplementation

**Reviewer** #1

**Comment:** L. 78-9: Delete the second sentence starting with “Despite all” because it is redundant.

**Response**: Its effect remained the major health problems of the communities in Ethiopia which indeed requires identification of pockets for further policy for intensified implementation directions.

**Reviewer** #1

**Comment:** L. 86: Delete “Ethiopian Demographic Health survey” because it is already defined.

**Response**: The approval letter to use the dataset was obtained from the DHS and the dataset

**Reviewer** #1

**Comment:** L. 93: “collected”

**Response**: EDHS Collected data on fundamental traits of family health, including immunization…

**Reviewer** #1

**Comment:** L. 144: “is” should be “are”

**Response**: In this analysis, we used secondary data that are publicly available from EDHS

**Reviewer** #1

**Comment:** L. 159: Define “ANC” here.

**Response**: Over half (58.10%) of the respondents reported no ANC visit (ANC is the care that mother should obtain during pregnancy),

**Reviewer** #1

**Comment:** L. 163: “.” At the end of the sentence.

**Response**: They were also not working (71.69%) during data collection time.

**Reviewer** #1

**Comment:** 170: Ethiopia.

**Response**: This analysis showed that geospatial distribution of vitamin A supplementation was non-random in Ethiopia

**Reviewer** #1

**Comment:** L. 174:“northern”

**Response**: The local (Getis-Ord Gi*) statistics indicated that Tigray, Benshangul, Gambella, northern Afar, Eastern Oromia were identified as hotspot area; whereas SNNP, central part of Afar, southern Oromia, Somali, and border of Amhara were identified as cold spots region of the country

**Reviewer** #1

**Comment:** L. 175: “areas”

**Response**: The local (Getis-Ord Gi*) statistics indicated that Tigray, Benshangul, Gambella, norther Afar, Eastern Oromia were identified as hotspot areas; whereas SNNP, central part of Afar, southern Oromia, Somali, and border of Amhara were identified as cold spots region of the country

**Reviewer** #1

**Comment:** L. 176: “cold spot regions”

**Response**: The local (Getis-Ord Gi*) statistics indicated that Tigray, Benshangul, Gambella, norther Afar, Eastern Oromia were identified as hotspot area; whereas SNNP, central part of Afar, southern Oromia, Somali, and border of Amhara were identified as cold spots regions of the country

**Reviewer** #1

**Comment:** L. 180 and 181 and 197: Capital “A”

**Response**: vitamin a was changed vitamin A

**Reviewer** #1

**Comment:** Table 2: No need to capitalize “variance”.

**Response**: Community-level variance

**Reviewer** #1

**Comment:** L. 204: Delete “were”

**Response**: ……literacy status, religion, wealth status, birth order, and region became significant both separately and in mixed effect model.

**Reviewer** #1

**Comment:** L. 213-4: “Women who had worked during the data survey period were 1.4 times more likely to…”

**Response**: Women who had worked during data survey period were 1.40 times more likely to supplement vitamin A to their children with AOR of 1.40[1.20-1.50].

**Reviewer** #1

**Comment:** L. 237: “no” should be “not”

**Response**: The respondents’ wealth index was low. In statistical analyses, this was also accompanied by the significant association of lack of current work for the respondent which is connected with low vitamin A supplementation. Evidence shows that wealth index and work status are the independent predictors of vitamin A supplementation; however, there is not enough evidence to support wealth index in our study.

**Reviewer** #1

**Comment:** L. 239: Instead of “not learned” use “were uneducated”

**Response**: In our study, 66.38% of respondents were not educated, while those who learned…

**Reviewer** #1

**Comment:** L. 261: Delete “was”

**Response**: Vitamin A supplementation analysis also showed poor distribution in the Oromia region which is consistent with cumulative decreasing regional micronutrients coverage report from 2007 to 2011

**Reviewer** #1

**Comment:** L. 266-7: “More vitamin A supplementation problems were found in many regions, which still infers a deep-rooted issue in Ethiopia.”

**Response**: Accepted

**Comment:** L. 275: Delete the first “was”

**Response**: Overall the vitamin A supplementation in Ethiopia has remained below par and the distribution of risk followed the contextual nature of the regions

**Reviewer** #1

**Comment:** Reference 3 should be “Ross DA”.

**Response**: Ross DA., Recommendations for Vitamin A Supplementation, The Journal of Nutrition, Volume 132, Issue 9, September 2002, Pages 2902S–2906S,

**Reviewer** #1

**Comment:** Some titles have all capital letters and others do not.

**Response**: all references were checked and corrected for spelling and capitalization

**Reviewer** #1

**Comment:** Reference 5 and 7 and 17 and 19 and 20 do not have a journal.

**Response**: name of the journal were added to the references

**Reviewer** #1

**Comment:** Reference 14 needs a capital “World Bank”.

**Response**: World Bank Group. Ethiopia Nutrition project performance Asessment Report. 2019;(136172):9. Available from:

**Reviewer** #1

**Comment:** Reference 18 has complete names and not initials. Reference 27 is the same as 18

**Response**: Semba RD, Pee SD, Sun K, Bloem MW, Raju VK, Coverage of the National Vitamin A Supplementation Program in Ethiopia, Journal of Tropical Pediatrics, Volume 54, Issue 2, April 2008, Pages 141–144, https://doi.org/10.1093/tropej/fm.

**Reviewer** #1

**Comment:** Figures 2 and 3 and 4: “A” needs to be capitalized on the figure.

**Response**:

**Reviewer** #2

**Comment:** reviewing the English

**Response**: the whole document was reviewed for English with local professional editor

**Reviewer** #2

**Comment:** in depth description of the delivery model used for vitamin A supplementation

**Response**: In Ethiopia, vitamin A distribution followed three strategies: enhanced outreach strategy, community health days, and the routine health extension program (regular community health workers)

The supplementation of vitamin A in Ethiopia follows some strategic modalities like enhanced outreach strategy, community health days, and the routine health extension program. Enhanced outreach strategy was integrated in to the semi-annual or quarterly campaigns of deworming and malnutrition screening for implementation. Community health day is the campaign that has been organized at lower administrative (Kebele) levels. Community health day has been integrated in to the health extension program. Indeed currently, vitamin A supplementation is considered as the component of health extension program and majorly being disseminated by health extension (community health workers).

The increase from previous studies prevalence might be due the make shift in strategies of delivery of vitamin A and integration of vitamin A delivery system in to health extension program
